# Treatment of Water Contaminated with Non-Steroidal Anti-Inflammatory Drugs Using Peroxymonosulfate Activated by Calcined Melamine@magnetite Nanoparticles Encapsulated into a Polymeric Matrix

**DOI:** 10.3390/molecules27227845

**Published:** 2022-11-14

**Authors:** Reza Darvishi Cheshmeh Soltani, Fatemeh Asgari, Negin Hassani, Yeojoon Yoon, Alireza Khataee

**Affiliations:** 1 Department of Environmental Health Engineering, School of Health, Arak University of Medical Sciences, Arak 3819693345, Iran; 2 Department of Environmental and Energy Engineering, Yonsei University, Wonju 26493, Korea; 3 Research Laboratory of Advanced Water and Wastewater Treatment Processes, Department of Applied Chemistry, Faculty of Chemistry, University of Tabriz, Tabriz 5166616471, Iran; 4 Department of Environmental Engineering, Gebze Technical University, 41400 Gebze, Turkey

**Keywords:** catalyst, iron oxide nanoparticles, sodium alginate, microencapsulation, peroxymonosulfate, pharmaceuticals

## Abstract

In the present study, calcined melamine (CM) and magnetite nanoparticles (MNPs) were encapsulated in a calcium alginate (CA) matrix to effectively activate peroxymonosulfate (PMS) and generate free radical species for the degradation of ibuprofen (IBP) drug. According to the Langmuir isotherm model, the adsorption capacities of the as-prepared microcapsules and their components were insignificant. The CM/MNPs/CA/PMS process caused the maximum degradation of IBP (62.4%) in 30 min, with a synergy factor of 5.24. Increasing the PMS concentration from 1 to 2 mM improved the degradation efficiency from 62.4 to 68.0%, respectively, while an increase to 3 mM caused a negligible effect on the reactor effectiveness. The process performance was enhanced by ultrasound (77.6% in 30 min), UV irradiation (91.6% in 30 min), and electrochemical process (100% in 20 min). The roles of O•H and ${\mathrm{SO}}_{4}^{•-}$ in the decomposition of IBP by the CM/MNPs/CA/PMS process were 28.0 and 25.4%, respectively. No more than 8% reduction in the degradation efficiency of IBP was observed after four experimental runs, accompanied by negligible leachate of microcapsule components. The bio-assessment results showed a notable reduction in the bio-toxicity during the treatment process based on the specific oxygen uptake rate (SOUR).

## 1. Introduction

The presence of pharmaceutical compounds in water resources causes high ecological risks to certain living organisms [1,2]. Among them, ibuprofen (IBP) poses chronic ecological risks for high organisms such as fish [3]. It is an extensively consumed nonsteroidal anti-inflammatory drug (the world’s third most consumable pharmaceutical compound) [4]. It is extensively used for the treatment of inflammatory rheumatic diseases, muscular pains, toothache, headache and cold fever [5,6,7]. The presence of IBP poses a major risk to human health and disturbs the balance of aquatic ecosystems. Due to its extensive applications, large amounts of IBP are produced annually, while its major fraction is excreted unmetabolized via urine and feces after consumption [4]. Therefore, the removal and degradation of IBP from aquatic environments is an environmental and health necessity. Hydroxyl radical (O•H)-based advanced oxidation processes (AOPs) have been successfully utilized for the degradation of refractory organic pollutants [8,9,10]. Among them, Fenton-based processes [11], photocatalysis [12,13,14] and catalytic ozonation [15] have been developed and used to effectively decompose IBP. 

Recently, sulfate radical (${\mathrm{SO}}_{4}^{•-}$)-based AOPs have gained more attention especially for the decomposition of refractory organic contaminants because of higher standard redox potential (2.5 to 3.1 V vs. NHE) of sulfate radical in comparison with the hydroxyl radical (1.8 to 2.7 V vs. NHE) [16,17,18,19]. In this regard, the activation of peroxymonosulfate (PMS), as a main precursor of sulfate radicals, has been proposed as the main procedure to generate sulfate radicals in water [18,20]. Several physical and chemical agents such as metal ions [21,22], carbonaceous compounds [23,24], ultrasound (US) [16,25] and ultraviolet (UV) light [26] are utilized for the activation of PMS. Based on the type of activating agent, both sulfate and hydroxyl radicals are generated through the following reactions [27,28]:

(1) $${\mathrm{HSO}}_{5}^{-}\overset{\mathrm{Metal}\;\mathrm{oxides}\;\mathrm{and}\;\mathrm{composites}\;}{\to }{\mathrm{SO}}_{4}^{•-}+{\mathrm{OH}}^{-}$$

(2) SO4•−+H2O→SO42−+O•H+H+

(3) HSO5−→US, UV and heat SO4•−+O•H

The activation of PMS by the transition metals oxides has attracted significant attention. Magnetite nanoparticles (MNPs) are efficiently utilized for the activation of PMS to produce free sulfate radicals [25,29,30]. Carbocatalysts such as activated carbon, biochar and graphitic carbon nitride have also been applied for the metal-free activation of PMS because of high cost-effectiveness, high surface area and low generation of secondary pollutants [23,24,31]. 

The excellent activity of the carbocatalysts, especially N-contained species, in the activation of PMS via a non-radical route has been verified. This has led to increased attempts to improve the N functionality of the carbocatalysts [24,32]. It has been reported that the incorporation of N into the sp^2^ carbon network and surface functionalization results in enhanced PMS activation [18]. In the present study, based on our hypothesis, calcined melamine (CM) was applied as an N-containing compound for the nonradical-based activation of PMS. It has been shown that the incorporation of N-rich compounds such as melamine into another activating agent results in the enhanced activation of PMS [24,33]. The development of nitrogen/carbon/transition metals as composite catalysts with dispersed reactive sites has been proposed as an attractive approach for the environmental remediation [34,35,36,37]. The CM microstructure can act as a support for the immobilization of the MNPs. The washout of nanoparticles from the reactor is a major environmental and economic concern [38]. The release of fine particles of the catalyst into the environment during PMS-based treatment processes has been reported [18,24]. Therefore, in the present study, MNPs were incorporated into the CM microstructure to obtain an efficient composite for the intensified activation of PMS. To vigorously inhibit the release of nanoparticles and to improve the ability of the treatment process to be operated in consecutive operational runs, the encapsulation of both MNPs and CM into the alginate matrix as a natural polymer was considered. Overall, the main aim of the present investigation was the incorporation of MNPs into the CM microstructure and their encapsulation in the alginate matrix to efficiently activate PMS for the degradation of IBP in aquatic solutions. 

## 2. Materials and Methods

### 2.1. Chemicals 

IBP (formula: C_13_H_18_O_2_; molecular weight: 206.29 g/mol; purity ≥ 98%) was purchased from Sigma-Aldrich Co., St. Louis, MO, USA. Analytical grade PMS (HSO_5_^–^) was obtained from Merck Co. (Darmstadt, Germany) and used as a sulfate radical precursor. Sodium alginate powder from *Laminaria hyperborean* was prepared by BDH Co. (Poole, UK), GB and used for the encapsulation. All other chemicals and reagents were purchased from Merck Co. (Germany).

### 2.2. Synthesis Procedure

MNPs were obtained by anodic corrosion in an electrochemical reactor equipped with an iron anode [39]. To obtain CM, melamine waste was calcined in an electric furnace set to a temperature of 500 °C for 2 h. The obtained powder was cooled, washed repeatedly with deionized water, and dried in an oven at 80 °C before use. Then, specified amounts of sodium alginate powder (2 g) were added to 100 mL of distilled water and magnetically agitated to achieve homogeneity. Afterwards, 1 g MNPs and 1 g CM powder were simultaneously added to the as-prepared viscose solution of sodium alginate and magnetically mixed to obtain a homogeneous mixture. The resulting mixture was added to 0.5 M calcium chloride solution using an insulin syringe. Consequently, CM and MNPs were encapsulated in the calcium alginate matrix, forming spherical beads of uniform size. The beads were separated and washed with distilled water. Finally, they were dried at ambient temperature, ground and stored in a desiccator for the PMS activation. 

### 2.3. Experiments

The degradation of IBP by the CM/MNPs/CA/PMS process was performed in a 400 mL batch flow-mode cylindrical glass experimental reactor. Double distilled water was used for the preparation of IBP-contained bulk solution of the reactor. Firstly, role of the adsorption process in the removal of IBP was determined at an IBP concentration of 25 µM, a CM/MNPs/CA dosage of 0.3 g/L and a PMS concentration of 1 mM within the equilibrium time of 30 min. Then, the role of PMS-based processes in the removal of IBP was determined and compared under the same operating conditions. The reaction rate of the IBP degradation was determined using a pseudo-first order kinetic equation. The effect of PMS concentration (0.5−3 mM), scavenging compounds (0.1 M) and co-compounds (0.01 M) on the reactor effectiveness was examined. A 6-W UVC lamp was applied to enhance the treatment process. Moreover, an ultrasonic bath (Elma, P30H, Singen, Germany) with a frequency of 80 kHz was utilized in order to improve the process performance. For the electrochemical enhancement, Ti/Ru anode and graphite cathode were placed in the CM/MNPs/CA/PMS reactor containing 0.05 M sodium sulfate as the working electrolyte. The anode and cathode electrodes were connected to a DC power supply adjusted at a constant current intensity of 100 mA. All experiments were performed at least twice and mean values were used for the data presentation.

### 2.4. Analysis

First, 2-mL samples were withdrawn from the reactor and centrifuged (10,000 rpm for 5 min) to prepare particle-free samples for high-performance liquid chromatography (HPLC) to quantify the remaining concentration of IBP after the treatment. The oxidizing ability of PMS was quenched before HPLC analysis by adding ethanol (0.5 mL) to each sample. A UV detector (230 nm) and an RP amide column were used for the detection. Acetonitrile and phosphate buffer (pH: 3.5) at a volumetric ratio of 55:45 were mixed and used as the mobile phase. The sample injection rate was 1 mL min^−1^. Moreover, the remaining concentration of PMS after treatment with CM/MNPs/CA/PMS was measured using a modified iodometric titration method employing a UV-visible spectrophotometer at a wavelength of 395 nm [30,40]. 

Change in the structure of IBP and its conversion to inorganic species during the treatment process of CM/MNPs/CA/PMS was checked utilizing a Skalar TOC analyzer (Breda, The Netherlands). Scanning electron microscopy (SEM) images were used to assess the surface morphology of the samples (TESCAN, Mira3, Brno, Czech Republic). The SEM microscope was equipped with an energy dispersive X-ray (EDX) spectrometer to determine the elemental composition of the samples. The elemental distribution was determined by elemental mapping. The surface structure of the samples and their functional groups were characterized by Fourier transform infrared (FTIR) spectroscopy (Bruker, Rheinstetten, Germany). The crystallographic composition of the samples was determined using X-ray diffraction analysis (XRD) (Tongda, model: TD-3700, Shanghai, China). The spectral properties and band gap energies of the samples were determined using differential reflectance spectroscopy (DRS) (Analytik Jena, S250, Jena, Germany). 

The concentration of mixed liquor volatile suspended solids (MLVSS) of the activated sludge sample was measured for the bio-toxicity assessment [41]. An electric furnace adjusted at 550 °C was used for the analysis. The volatile portion of the activated sludge was used as an indication of the active microorganisms. Using MLVSS analysis data, the bio-toxicity examination was performed based on the specific oxygen uptake rate (SOUR), calculated by dividing OUR by MLVSS as mg O_2_/mg MLVSS.d, before and after the treatment process [38]. 

## 3. Results and Discussion

### 3.1. Characterization 

**Figure 1:**
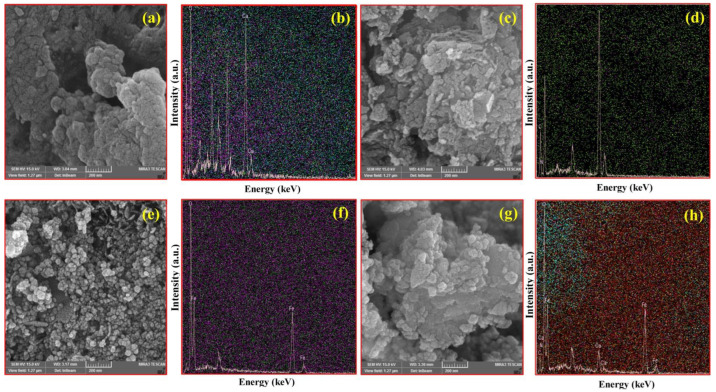
SEM images of CA (**a**) with EDX-Map (**b**), CM (**c**) with EDX-Map (**d**), MNPs (**e**) with EDX-Map (**f**), and CM/MNPs/CA (**g**) with EDX-Map (**h**).

SEM images of CA, CM, MNPs, and CM/MNP/CA are shown in Figure 1. The surface morphology of the CA is shown in Figure 1a. Figure 1b shows an EDX micrograph of CA, presenting its elemental composition; as shown, it was mainly composed of Ca, C, and O elements. Figure 1c exhibits the scaly structure of the CM with the defect sites. This structure improves the surface area and, consequently, the reactive sites of the CM for the PMS activation. By using EDX mapping, a uniform distribution of CM elements, especially N and C, can be seen in Figure 1d. The presence of these elements is favorable for PMS activation. The SEM images of MNPs showed nanostructures with uniform spherical shapes (Figure 1e). The presence and distribution of Fe and O are shown in Figure 1f. Accordingly, the EDX micrograph was composed of Fe and O element peaks, indicating the high purity of the electrochemically synthesized MNPs. The surface morphology of CM/MNPs encapsulated in the CA polymeric matrix is shown in Figure 1g. In this SEM image, nanostructures can be clearly observed even after encapsulation in the CA matrix. This indicates that the encapsulated components of CM and MNPs were exposed to PMS molecules for the efficient activation. The CM and MNPs elements, along with the constituent elements of CA, can be found in the composition of the as-prepared microcapsules (Figure 1h). Representative SEM images of CA, CM, MNPs, and their combination, along with the corresponding elemental distributions, are shown in Figure S1.

**Figure 2:**
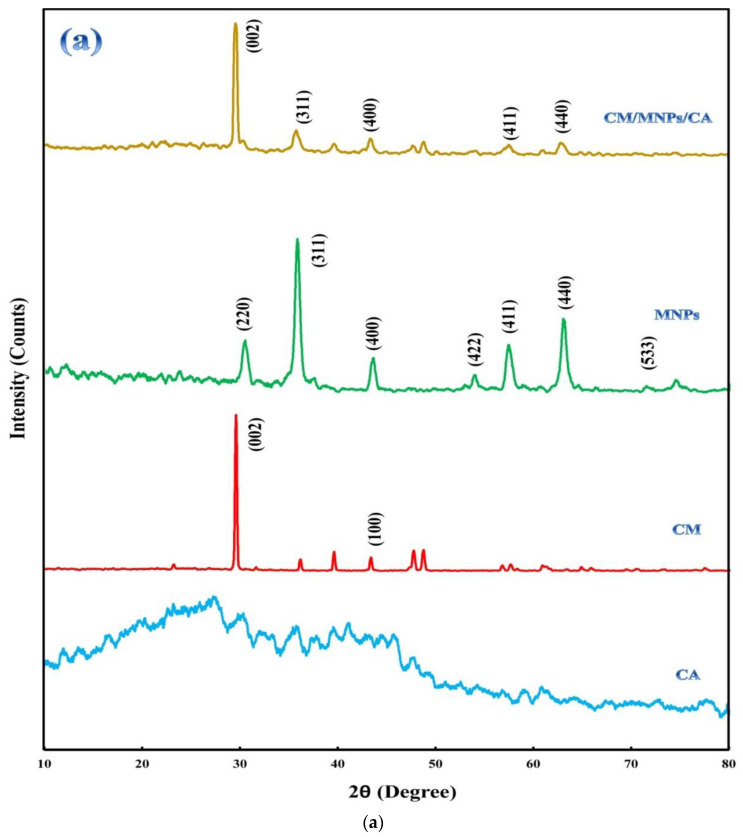

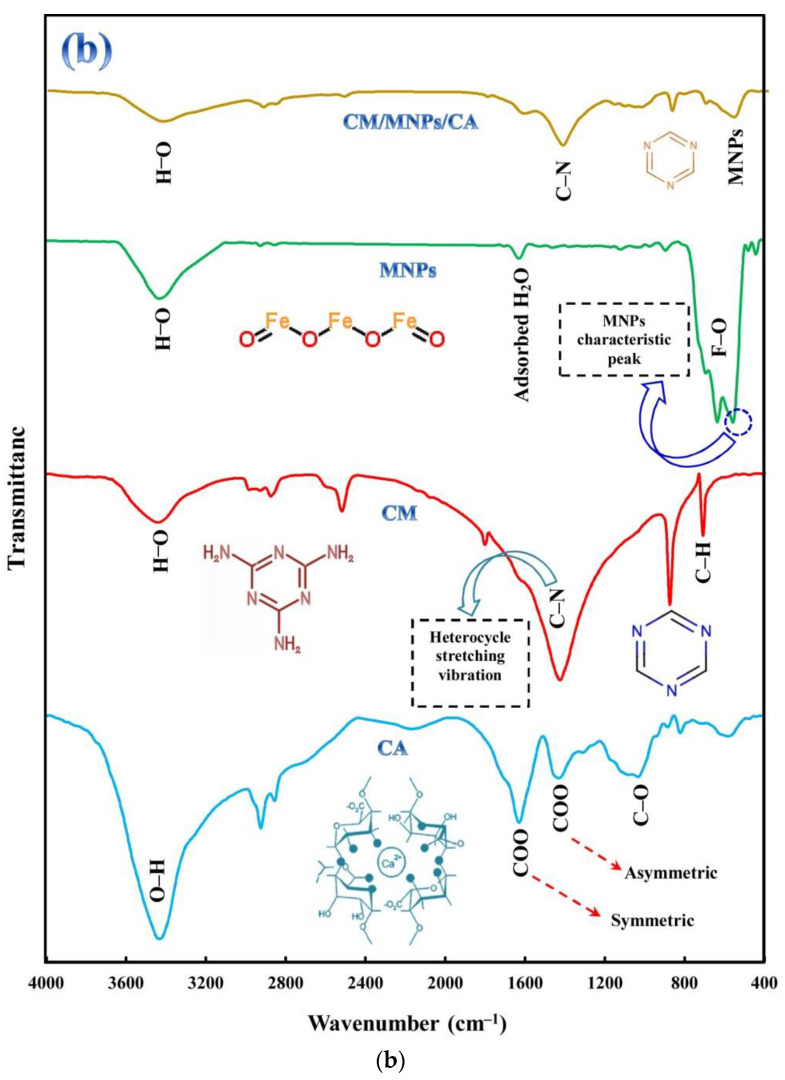
XRD patterns of CA, CM, MNPs and their encapsulated form (**a**), along with FT-IR spectra of CA, CM, MNPs and CM/MNPs/CA microcapsule (**b**).

XRD patterns of the samples are shown in Figure 2a, revealing a crystalline structure and the component composition. The diffractogram of CA indicated its amorphous nature in comparison with CM and MNPs. In the diffractogram of CM, the presence of main peaks at 29.50°and 43.28°, ascribed to the (002) and (100) planes, indicates crystalline carbon with a strong interplanar structure [21,42]. This may be attributed to the interlayer stacking of the stratified structure produced via the self-polymerization of melamine together with its graphitic structure [18,21]. The peaks located at 30.30°, 35.74°, 43.46°, 53.76°, 57.32°, 62.94°, and 74.32°are related to the (220), (311), (400), (422), (511), (440), and (533) planes of the magnetite composition, respectively. These peaks and their positions match JCPDS card number 19-0629 [30,43]. The presence of the abovementioned sharp peaks indicates the high purity of the electrochemically synthesized MNPs with a significant degree of crystallization. As it is obvious, the encapsulation of CM and MNPs did not influence their crystalline structure. The position of the peaks related to CM and MNPs did not change after encapsulation.

FTIR analysis was performed on powdered samples using the KBr pellet method. The FTIR spectra of the samples are shown in Figure 2b. The FTIR spectrum of the CA showed four distinct peaks at 1080, 1465, 1645, and 3462 cm^−1^, which were associated with C-O, asymmetric stretching vibration of COO, and symmetric stretching vibration of COO and OH bonds, respectively [44,45]. For the CM powder, the sharp band between 1200 and 1700 cm^−1^ can be ascribed to C-N heterocycle stretching vibration [21]. The sharp peaks located at 653 and 871 cm^−1^ are related to the bending vibration of C-H and the bending vibration of the triazine ring of melamine, respectively [42,46]. The FTIR spectrum of the MNPs shows two main peaks at 1660 and 3475 cm^−1^ indicating the stretching vibration of absorbed water and OH groups, respectively [43]. The peaks located at 576 and 646 cm^−1^ are related to the vibration of the Fe-O band in the surface structure of MNPs. The peak at 576 cm^−1^ was specified as the characteristic peak of magnetite [47]. As shown in Figure 2a, the characteristic peaks of CM and MNPs were observed in the spectrum of the CM/MNPs/CA microcapsule. However, changes in the intensity of surface functional group peaks after encapsulation can be clearly observed in the CM/MNPs/CA microcapsule spectrum, suggesting the involvement of the aforementioned bonds in the immobilization and encapsulation of composing components. 

**Figure 3:**
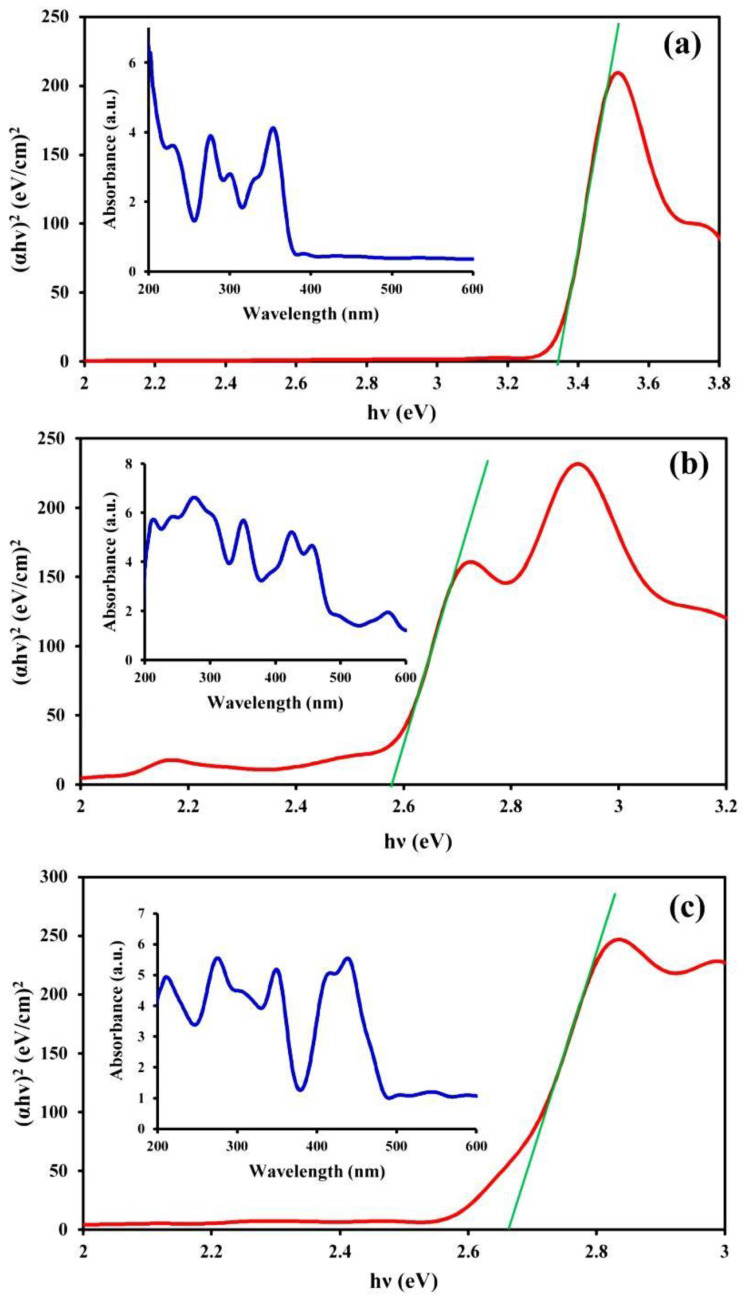
DRS spectra of CM (**a**), MNPs (**b**) and CM/MNPs/CA (**c**) along with the corresponding band gap energies.

DRS spectroscopy was employed to assess the optical properties of the samples and, consequently, their catalytic activities (Figure 3). This analysis was also useful for determining the structure of the samples. Figure 3a shows the presence of two main adsorption peaks in the UV-wavelength region for CM, while in the case of the MNPs, three other peaks were observed in the visible light wavelength region (400–600 nm) in addition to the UV-wavelength region peaks (Figure 3b). The peaks in the visible light wavelength region indicated the presence of transition metal ions. Peaks associated with both CM and the MNPs were also observed in the spectrum of the CM/MNPs/CA microcapsule (Figure 3c), which indicated the existence of CM and MNPs in the composition of the microcapsule. Overlapping of the peaks in the microcapsule spectrum implies the effective interaction of CM, MNPs, and CA, generating integrated microcapsules. The band gap energy (*E_g_*) was also calculated using Planck’s constant (*h*), the optical absorption index (*n*), the absorption coefficient (*α*), and the frequency of light radiation (*ν*) using the following equation:

(4) $$\alpha hv=A{\left(hv-E_{g}\right)}^{\frac{n}{2}}$$

The *E_g_* values of the pure CM, pure MNPs, and CM/MNP/CA microcapsules were 3.34, 2.58 and 2.67 eV, respectively. The higher the *E_g_*, the lower the catalytic activity. The low value of *E_g_* obtained for the CM/MNP/CA microcapsule in comparison with that of pure CM indicates its high catalytic activity even after the encapsulation. On the other hand, the combination of CM and MNPs in the CA matrix not only did not significantly affect the catalytic activity of the components, but it also improved the catalytic performance in comparison with the pure CM.

### 3.2. Adsorption Role

The adsorption process may have interfered with the removal of IBP by the CM/MNPs/CA/PMS process. For this reason, adsorption of IBP onto the MNPs, CM and CM/MNPs/CA was evaluated using the same dosage of aforementioned compounds (0.3 g/L) at the initial IBP concentration of 25 µM under natural pH conditions. The amount of IBP (µM) adsorbed onto the adsorbents (g) was determined using the following equation [48]:

(5) $$Adsorption=\frac{\left(C_{0}-C_{e}\right)\times V}{M}$$

where *C*_0_ and *C_e_* represent the initial and remaining concentrations of IBP in the reactor (µM), respectively. In addition, *V* (L) and *M* (g) indicate the working volume and dosage of the adsorbent, respectively. The adsorption results are presented in Figure S2. The adsorption capacity of CM/MNPs/CA was the highest in comparison with the sole use of MNPs or CM. To estimate the maximum adsorption capacity of the samples (*q_m_*, µM/g) toward IBP removal, the Langmuir isotherm modeling was implemented using the following equation [48,49,50]:

(6) $$\frac{C_{e}}{q_{e}}=\frac{1}{kq_{m}}+\frac{1}{q_{m}}C_{e}$$

where *q_e_* (µM/g) and *K* (L/µM) indicate the amount of IBP adsorbed at equilibrium and the affinity of the adsorbent toward IBP, respectively. Figure S3 shows the results of the Langmuir isotherm model. The obtained correlation coefficients demonstrated the suitability of Langmuir isotherm modeling to describe the adsorption process of IBP onto the MNPs (*q_m_*: 0.04 µM/g), CM (*q_m_*: 0.20 µM/g) and CM/MNPs/CA (*q_m_*: 0.35 µM/g). Based on the obtained maximum adsorption capacities, it may be concluded that the adsorption process plays a minor role in the removal of IBP from the aquatic phase.

### 3.3. Comparison of PMS-Based Treatment Processes

**Figure 4:**
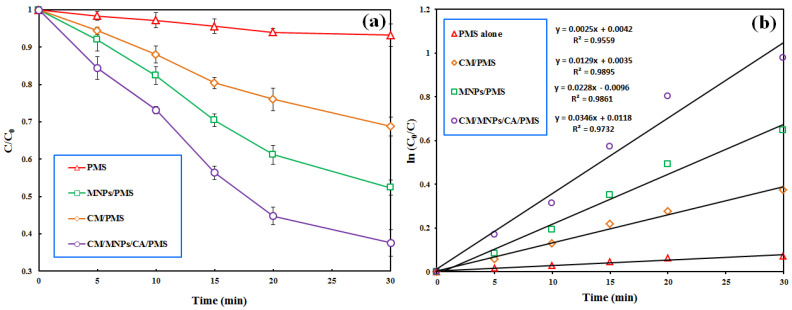
Effectiveness of various PMS-based treatment processes involved in the removal of IBP (**a**) accompanied with the results of pseudo-first order kinetic modeling (**b**). Experimental conditions: [IBP]_0_: 25 µM, catalyst dosage: 0.3 g/L, [PMS]_0_: 1 mM, reaction time: 30 min.

The results, depicted in Figure 4a, show the negligible effectiveness of PMS alone in the removal of IBP from the reactor within a reaction time of 30 min (6.8%). The direct degradation of the target pollutant by the PMS alone was performed via a non-radical oxidation mechanism [51]. According to similar studies, PMS alone is not effective in the degradation of organic pollutants [5,17,37]. Moreover, the negligible decomposition of IBP by the PMS alone implies that PMS is selective toward specific organic pollutants. This result also indicates that neither ${\mathrm{SO}}_{4}^{•-}$ nor O•H participated in the decomposition of the target pollutant by the PMS alone. The utilization of PMS in the presence of CM and MNPs led to the degradation efficiencies of 31.2 and 47.6% in 30 min, respectively. This finding indicates that MNPs have more activation potential than CM for the PMS activation. Similar results were reported by Bicalho et al. (2020) in their study on the degradation of acetaminophen using PMS activated by an iron waste/graphitic carbon nitride composite [21].

In the case of CM, the incorporation of N into the sp^2^ carbon network and the surface functionalization of CM resulted in the activation of PMS. CM contains large amounts of N for the PMS activation [24]. Many previous studies have reported that N-rich compounds provide an effective degradation reaction via a non-radical mechanism [52,53]. Nitrogen modulates the electronic characteristics of the carbon lattice by disturbing the spin density and electron distribution [53]. Using MNPs, PMS activation was carried out based on the following equations [25]:

(7) $$\mathrm{Fe}\left(\mathrm{III}\right)+{\mathrm{HSO}}_{5}^{-}\to \mathrm{Fe}\left(\mathrm{II}\right)+{\mathrm{SO}}_{5}^{•-}+H^{+}$$

(8) $$\mathrm{Fe}\left(\mathrm{II}\right)+{\mathrm{HSO}}_{5}^{-}\to \mathrm{Fe}\left(\mathrm{III}\right)+{\mathrm{SO}}_{4}^{•-}+{\mathrm{OH}}^{-}$$

Under the same operating conditions, a degradation efficiency of 62.4% was achieved when CM/MNPs/CA was used as the activating agent. The carbonaceous structure of CM containing nitrogen accompanied by MNPs created an appropriate structure with oxygen-containing surface functional groups for the activation PMS to generate free oxidizing radicals [23]. The incorporation of nitrogen into the carbon matrix increases the catalytic activity owing to its electronic adapting activity toward the topological carbonaceous structure. PMS can be activated using defect-rich carbon-based catalysts [35]. Surface-activated PMS is one of the major reactive species for the degradation of organic compounds via an electron transfer mechanism, as represented by the following equation:

(9) $$\mathrm{CM}-\pi^{-}+{\mathrm{HSO}}_{5}^{-}\to \mathrm{CM}-\pi +{\mathrm{SO}}_{4}^{•-}+{\mathrm{OH}}^{-}$$

The enhanced degradation efficiency of IBP was also related to the oxidation of magnetite (Fe_3_O_4_) containing both Fe(II) and Fe(III) while maintaining the inverse spinel crystal framework. The magnetite phase is formed via an internal reduction mechanism, while several interactions with highly reactive sites and surface functional groups occur on the CM surface in the microcapsule [21,30,54].

(10) CM/MNPs/CA+HSO5−→SO4•−+O•H

(11) SO4•−/O•H+IBP→Intermediate byproducts→CO2+H2O

In agreement with the present study, Wu et al. reported the effectiveness of iron@nitrogen-containing porous carbon catalysts for the activation of PMS to decompose bisphenol F in the aquatic phase [37]. Our results revealed a decrease in the PMS concentration from 1.0 to 0.06 mM after a treatment time of 30 min, indicating the consumption of PMS and its transformation to free radical species for the degradation of IBP. A pseudo-first-order kinetic model was successfully applied to describe the process data with a high correlation coefficient (R^2^ ≥ 0.9). Figure 4b displays the results of kinetic modeling for the PMS alone, CM/PMS, MNPs/PMS and CM/MNPs/CA/PMS processes with their reaction rate constants (*k*). The overall synergy for the process was computed using the following equation [7]:

(12) $$Synergyfactor=\frac{k_{CM/MNPs/CA/PMS}}{k_{CM/MNPs/CA}+k_{PMS}}$$

Accordingly, a synergy factor of 5.24 was obtained when considering the reaction rate constants of the adsorption process onto CM/MNPs/CA (*k_CM_*_/*MNPs*/*CA*_) and PMS alone (*k_PMS_*) versus the reaction rate constant of the combined process (*k_CM_*_/*MNPs*/*CA*/*PMS*_). 

### 3.4. Role of the Radical Species

**Figure 5:**
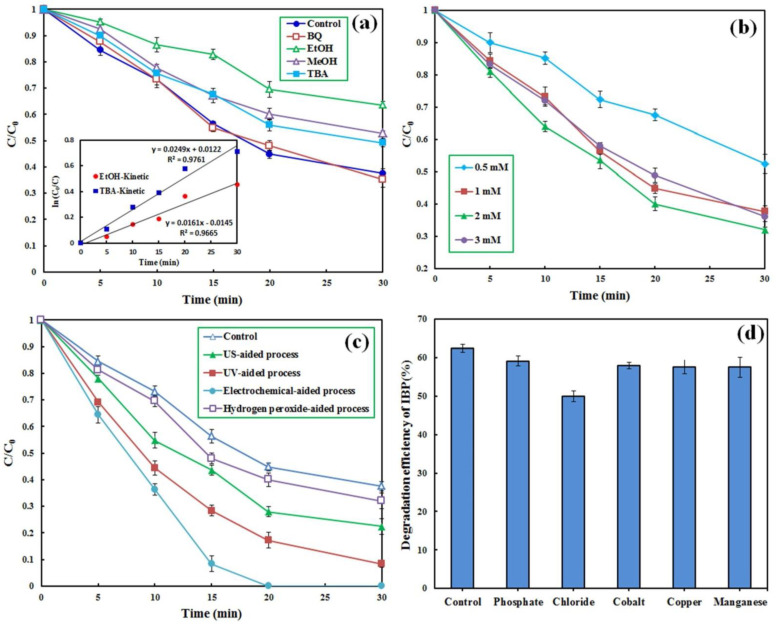
Effect of scavenging compounds (**a**), PMS concentration (**b**), enhancers (**c**) and presence of co-compounds (**d**) on the degradation of IBP by the CM/MNPs/CA/PMS process. Experimental conditions: [IBP]_0_: 25 µM, catalyst dosage: 0.3 g/L, reaction time: 30 min, [scavengers]_0_: 0.1 M, [H_2_O_2_]_0_: 50 mM, current intensity: 100 mA, [co–compounds]_0_: 0.01 M.

To determine the role of different radical species in the decomposition and conversion of IBP, scavenging compounds of ethanol (EtOH), methanol (MeOH), tert-Butyl alcohol (TBA), and benzoquinone (BQ) with a given concentration of 0.1 M were added to the PMS-based reactor. Figure 5a shows that the addition of BQ, a superoxide anion radical (O_2_^•−^) scavenger [42], did not significantly influence the reactor performance, indicating an insignificant role of this radical species in the degradation of IBP molecules. The addition of TBA and MeOH decreased the degradation efficiency of IBP from 62.4% to 50.5 and 47.2%, respectively. Both TBA and MeOH have a higher O•H scavenging potential than ${\mathrm{SO}}_{4}^{•-}$. Conversely, EtOH has a higher potential to scavenge ${\mathrm{SO}}_{4}^{•-}$ than O•H [55]. To determine the contribution of O•H and ${\mathrm{SO}}_{4}^{•-}$ to the degradation of IBP, the reaction rate constants for the degradation of IBP were estimated in the presence of EtOH and TBA based on the pseudo-first-order kinetic model. Accordingly, the following equations were applied to specify the role of the oxidizing radicals in the degradation of IBP:

(13) RoleofO•H=kCM/MNPs/CA/PMS−kTBAkCM/MNPs/CA/PMS×100

(14) $$Role\;of\;{\mathrm{SO}}_{4}^{•-}=\frac{k_{TBA}-k_{EtOH}}{k_{CM/MNPs/CA/PMS}}\times 100$$

Consequently, the roles of O•H and ${\mathrm{SO}}_{4}^{•-}$ in the decomposition of IBP by the CM/MNPs/CA/PMS process were 28.0 and 25.4%, respectively. This finding indicates the role of other mechanisms in the degradation of IBP, along with the O•H and ${\mathrm{SO}}_{4}^{•-}$ radicals. In a similar study, the main role of both O•H and ${\mathrm{SO}}_{4}^{•-}$ in the degradation of organic dyes by the magnetite-activated PMS process was demonstrated and reported [25]. Overall, it has been demonstrated that the application of non-metal activators, especially carbon-based activators, reduces the adverse effects of scavenging compounds [56]. 

The non-radical mechanism is based on the creation of outer-sphere complexes between PMS and CM in the microcapsule, improving the reactivity between the oxidant and pollutant [23]. Therefore, a non-radical mechanism by charge transfer can be proposed as one of the main mechanisms involved in the transformation and degradation of IBP by the CM/MNPs/CA/PMS process. The surface nitrogen sites of the CM, together with the carbon matrix, are mostly responsible for the degradation of IBP through a non-radical mechanism [34].

### 3.5. PMS Concentration Effect 

Overall, higher degradation efficiency, together with lower consumption of chemical agents, is preferred from both economic and environmental viewpoints. The PMS concentration was varied in the range of 0.5–3 mM to attain the optimal value of the oxidizing agent for the CM/MNPs/CA/PMS process. The results showed that decreasing the PMS concentration from 1 to 0.5 led to a considerable reduction in the degradation efficiency from 62.4 to 47.6% in 30 min (Figure 5b).

As can be seen, increasing the PMS concentration from 1 to 2 mM improved the efficiency from 62.4 to 68.0%, respectively, while an increase to 3 mM resulted in a negligible effect on the reactor effectiveness. The improved degradation of IBP with increasing PMS concentration from 1 to 2 mM was due to the fact that the availability of PMS was the rate-limiting factor controlling the generation of radicals at low PMS concentrations in which increasing PMS concentration resulted in the generation of more free radicals. With increasing PMS concentration, the number of reactive sites on the catalyst became the rate-limiting factor because of the fixed dosage of the CM/MNPs/CA composite. Under such conditions, the generation rate of free radicals is not dependent on the PMS concentration [43]. Considering the low oxidative potential of PMS alone (see Figure 4a), extra PMS dosage has no favorable influence on the degradation efficiency of IBP [55]. In addition, excessive PMS concentrations may scavenge the generated radical species as shown in the following equations:

(15) HSO5−+O•H→SO5•−+H2O

(16) $${\mathrm{HSO}}_{5}^{-}+{\mathrm{SO}}_{4}^{•-}\to {\mathrm{SO}}_{5}^{•-}+{\mathrm{SO}}_{4}^{2-}+H^{+}$$

In conclusion, at a PMS concentration of 3 mM, the degradation efficiency of IBP was not notably improved, implying that the reactor effectiveness was closely dependent on the number of reactive sites available on the CM/MNPs/CA microcapsule. However, increasing the PMS concentration did not have a considerable effect on the process efficiency when the reactive sites of the encapsulated catalysts were saturated [17].

### 3.6. Enhancing Strategies

#### 3.6.1. Hydrogen Peroxide Addition

Different enhancing approaches were used for the enhancement of the process performance. Firstly, H_2_O_2_ was added to the bulk solution of the reactor. Figure 5c shows that the addition of H_2_O_2_ (50 mM) to the CM/MNP/CA/PMS process slightly enhanced the reactor efficiency (<6%).

(17) $$\equiv \mathrm{Fe}\left(\mathrm{III}\right)+H_{2}O_{2}\to \equiv \mathrm{Fe}\left(\mathrm{II}\right)+{\mathrm{HO}}_{2}^{•}+H^{+}$$

(18) ≡Fe(II)+H2O2→≡Fe(III)+O•H+OH−

(19) CM+H2O2→CM++OH−+O•H

However, some H_2_O_2_ molecules may scavenge O•H, thereby reducing degradation efficiency [27,30]. 

#### 3.6.2. US, UV and Electrochemical Enhancement

The degradation efficiency increased from 62.4 to 77.6% when the CM/MNPs/CA/PMS process was operated in an ultrasonic bath adjusted to a frequency of 80 kHz. The main mechanisms are proposed in the following equations [25].

(20) HSO5−+)))→SO4•−+O•H

(21) ≡Fe(II)+HSO5−+)))→≡Fe(III)+SO42−+O•H

(22) $$\equiv \mathrm{Fe}\left(\mathrm{II}\right)-{\mathrm{OH}}^{-}+{\mathrm{HSO}}_{5}^{-}\to \equiv \mathrm{Fe}\left(\mathrm{III}\right)-\left(\mathrm{OH}\right){\mathrm{OSO}}_{3}^{-}+{\mathrm{OH}}^{-}$$

(23) $$\equiv \mathrm{Fe}\left(\mathrm{II}\right)-\left(\mathrm{OH}\right){\mathrm{OSO}}_{3}^{-}+{\mathrm{OH}}^{-}+)))\to \equiv \mathrm{Fe}\left(\mathrm{III}\right)-{\mathrm{OH}}^{-}+{\mathrm{SO}}_{4}^{•-}$$

According to the aforementioned equations, some PMS can be ultrasonically activated to form hydroxyl and sulfate radicals. In addition, PMS reacts with Fe(II) on the catalyst surface, generating hydroxyl and sulfate radicals under US irradiation, while the as-generated Fe(III) can be regenerated to Fe(II) by the PMS. A degradation efficiency of greater than 90% was achieved when the CM/MNPs/CA/PMS process was operated under UV light irradiation owing to the following equation:

(24) HSO5−+hν→SO4•−+OH−→SO42−+O•H

Notably, complete degradation of IBP was obtained within 20 min when the CM/MNPs/CA/PMS process was conducted in an electrochemical cell equipped with a Ti/Ru anode and a graphite cathode with a current intensity of 100 mA. This enhancement could be due to the Ti/Ru anode and the presence of PMS and sodium sulfate electrolyte in the electrochemical cell, as represented in the following equations:

(25) Anode reaction: Ti/Ru+H2O→Ti/Ru(O•H)+H++e−

(26) Effect of PMS: HSO5−+Electrical energy→SO4•−+O•H

(27) $$\mathrm{Effect}\;\mathrm{of}\;\mathrm{electrolyte}:{\;\mathrm{SO}}_{4}^{2-}+\mathrm{Electrical}\;\mathrm{energy}\to {\mathrm{SO}}_{4}^{•-}+e^{-}$$

### 3.7. Co-Existing Compounds Effect

The effect of different inorganic species on the degradation of IBP by the CM/MNP/CA/PMS process was evaluated to elucidate its performance under real conditions. Cations (copper, cobalt, and manganese) and anions (phosphate and chloride) were added to the reactor. Figure 5d shows the change in the degradation efficiency of IBP in the presence of different inorganic species. Notably, the addition of cations, such as copper, cobalt, and manganese, along with phosphate anions, led to insignificant reduction in the degradation efficiency (<5%). The maximum reduction in the degradation efficiency of IBP was observed in the presence of chloride ions (approximately 12%). The added chloride ions might react with hydroxyl and sulfate radicals, generating chlorine radical with lower redox potential [27]:

(28) Cl−+O•H→ Cl•+OH−

(29) $${\mathrm{Cl}}^{-}+{\mathrm{SO}}_{4}^{•-}\to {\;\mathrm{Cl}}^{•}+{\mathrm{SO}}_{4}^{2-}$$

Comparatively, phosphate anions can quench only small amounts of sulfate radicals, causing little adverse effect on the overall degradation efficiency [27,57]. 

### 3.8. Reusability, Stability, Mineralization and Bio-Assessment 

**Figure 6:**
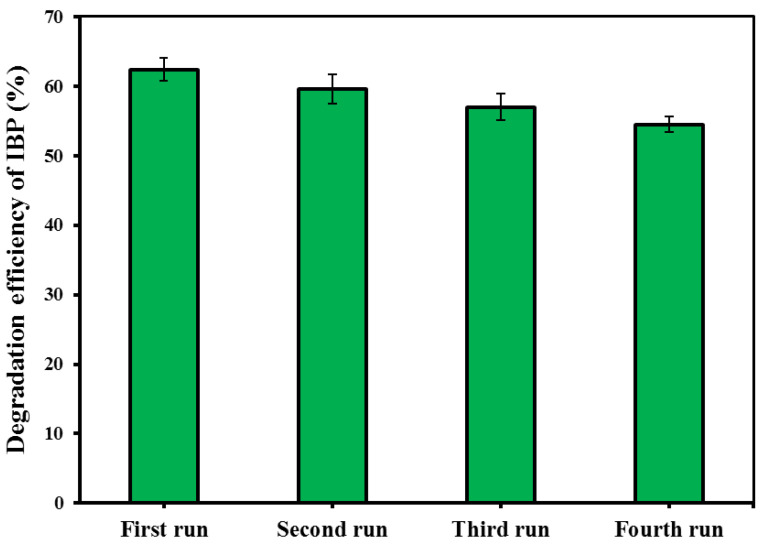
Reusability test results within four consecutive experimental runs.

Recycling of the spent catalyst in repeated experimental runs was considered to check the reusability potential of the CM/MNPs/CA microcapsules, as well as their economic feasibility. For this purpose, the spent CM/MNPs/CA was collected through centrifugation, washed with distilled water, and then dried in an oven for later use. The degradation efficiency of the IBP was determined using four consecutive experimental runs. According to the results, the degradation efficiency of IBP decreased from 62.4 to 54.5% at the end of fourth experimental run (Figure 6). This indicated an insignificant reduction in the process performance after four runs and, consequently, the high reusability potential of the CM/MNPs/CA microcapsules. This finding implies the suitability of microcapsules to use in real treatment systems. Indeed, the encapsulation of the catalyst nanoparticles improved the reusability potential as well as the possibility of separation for the next run.

The stability of the microcapsules was assessed by measuring Fe leachate during the process. The results showed a negligible release of Fe ions into the bulk solution (below the detection limit), even at the end of the fourth run. It has been shown that the encapsulation of iron oxide used for the activation of PMS not only prevents its leaching, but also promotes its catalytic activity [34]. 

The mineralization efficiency of IBP by the CM/MNPs/CA/PMS process was determined within a reaction time of 90 min. A mineralization efficiency of 23.2% was obtained after 90 min. The complete degradation of IBP was achieved within the same reaction time. In conclusion, the degradation of IBP results in the formation of organic intermediates, which might be difficult to mineralize to inorganic species, water, and carbon dioxide. To increase mineralization efficiency, longer reaction times and a combination of the main treatment process with other treatment techniques are needed [58]. 

A bio-toxicity test was performed on the process effluent to specify the toxicity of organic intermediates generated during the degradation of IBP as a parent compound. For this purpose, a microbial consortium of activated sludge was used as a living microbial population. Based on the obtained MLVSS concentration, the SOUR of the activated sludge-included effluent increased from 0.58 to 0.91 mg O_2_/mg MLVSS.d, indicating a lower bio-toxicity of the treated sample than the untreated sample. According to low mineralization efficiency, the results of bio-assessment indicated the formation of organic byproducts with lower toxicity for living organisms than the parent compound.

## 4. Conclusions

Co-microencapsulation of MNPs and CM powder was successfully performed in a CA polymeric matrix. The prepared microcapsules were effectively used for the activation PMS and, consequently, the degradation of IBP in water. Based on the results, both sulfate and hydroxyl radicals played a major role in the degradation of the target pollutant. The PMS concentration should be optimized to attain a cost-efficient treatment process with a lower release of chemicals into the ecosystem. The reusability of the prepared catalyst was considerable for the application in consecutive experimental runs with negligible loss of microcapsule components. The treatment process can be improved by conducting the process in an electrochemical cell, as well as by utilizing US and UV radiation. Moreover, the treatment process was not adversely influenced by the presence of inorganic species in real water samples. The degradation of IBP generated organic intermediates with a lower bio-toxicity for the microbial consortium. Therefore, the current treatment process could be applied as an effective and promising treatment technique with high stability and reusability potential for water resources contaminated by emerging pharmaceutical pollutants.

## Supplementary Materials

The following supporting information can be downloaded at: https://www.mdpi.com/article/10.3390/molecules27227845/s1, Figure S1. Representative SEM images of CA (a), CM (b), MNPs (c) and CM/MNPs/CA (d), along with elemental distribution; Figure S2. The amount of IBP adsorbed onto MNPs, CM and CM/MNPs/CA; Figure S3. Results of Langmuir isotherm modeling.
