# Supplementary material for: Treatment of Water Contaminated with Non-Steroidal Anti-Inflammatory Drugs Using Peroxymonosulfate Activated by Calcined Melamine@magnetite Nanoparticles Encapsulated into a Polymeric Matrix
Source: Molecules. 2022 Nov 14;27(22):7845. doi: 10.3390/molecules27227845 (PMC9698753; doi:10.3390/molecules27227845)
Supplement: Supplementary file 1 [file molecules-27-07845-s001.zip › molecules-1940096-supplementary.pdf]

Supporting information for

“Treatment of water contaminated with non-steroidal anti-inflammatory drugs using peroxymonosulfate activated by calcined melamine@magnetite nanoparticles encapsulated into a polymeric matrix”

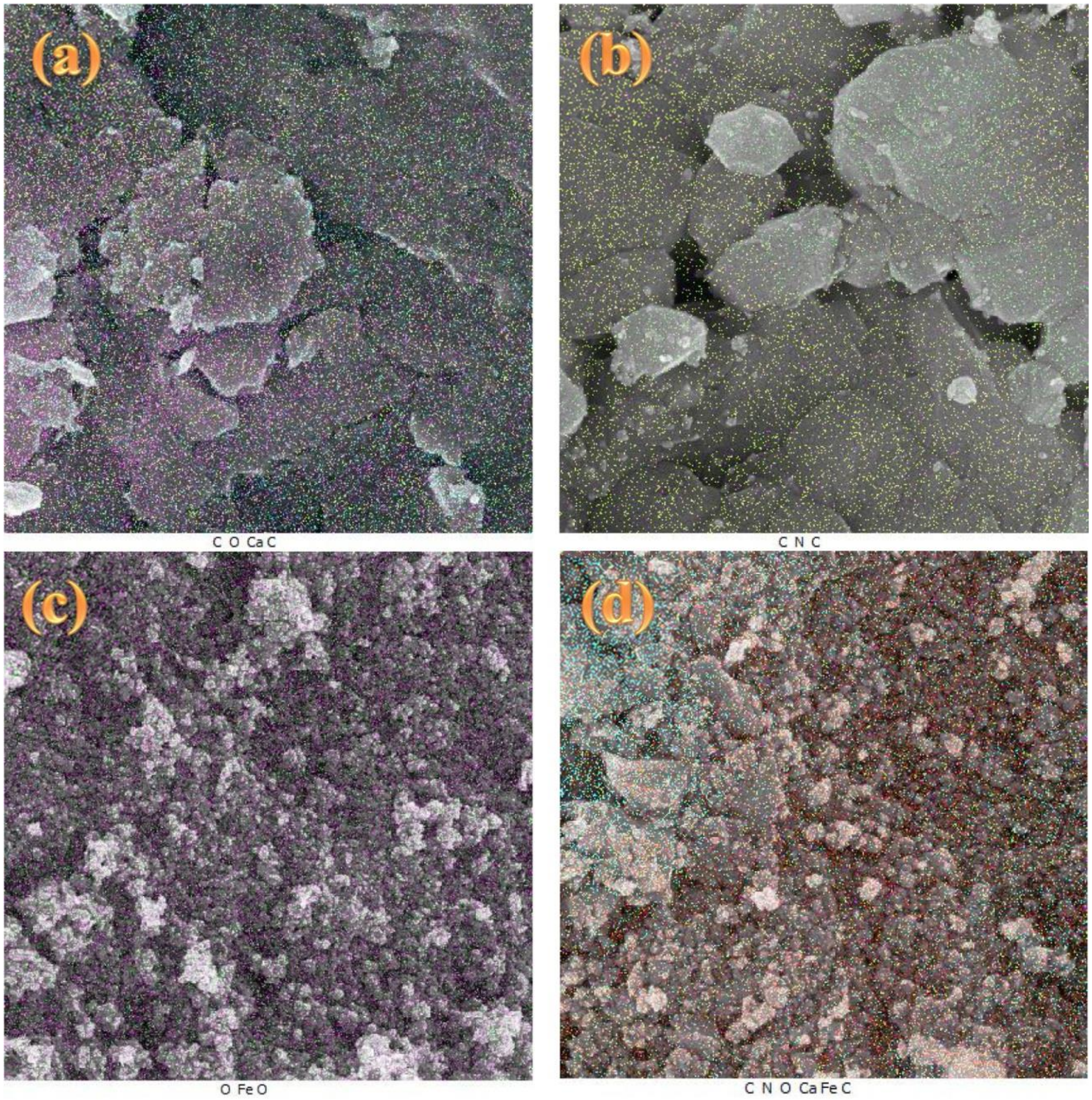

Figure S1. Representative SEM images of CA (a), CM (b), MNPs (c) and CM/MNPs/CA (d), along with elemental distribution.

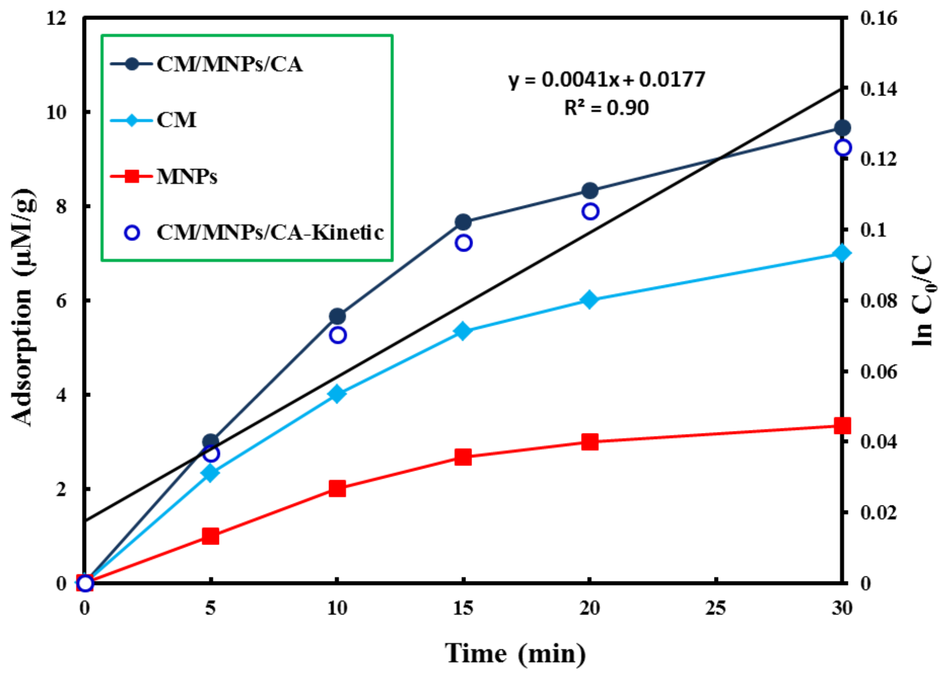

Figure S2. The amount of IBP adsorbed onto MNPs, CM and CM/MNPs/CA.

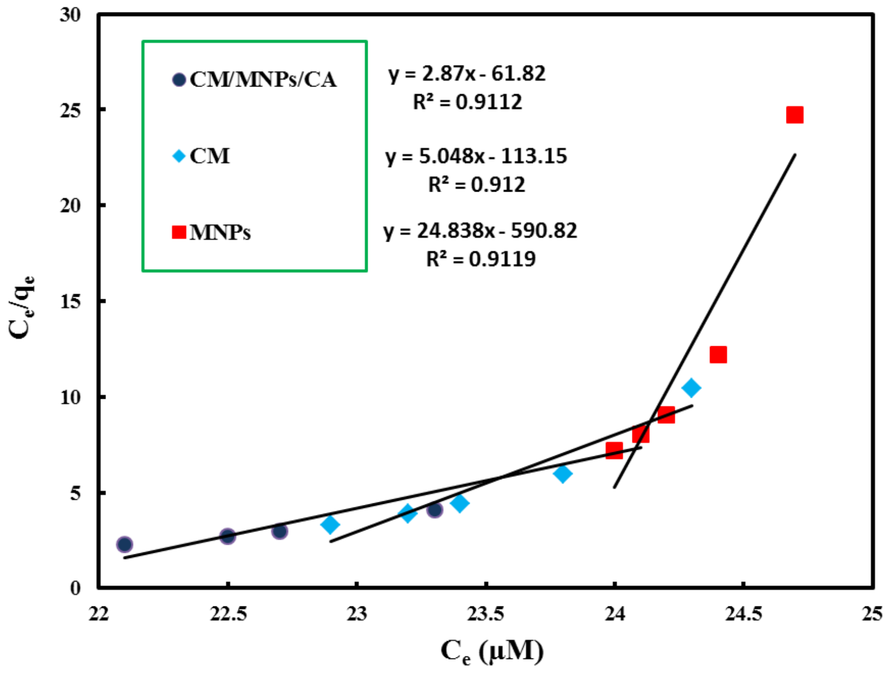

Figure S3. Results of Langmuir isotherm modeling.
